# GREAM: A Web Server to Short-List Potentially Important Genomic Repeat Elements Based on Over-/Under-Representation in Specific Chromosomal Locations, Such as the Gene Neighborhoods, within or across 17 Mammalian Species

**DOI:** 10.1371/journal.pone.0133647

**Published:** 2015-07-24

**Authors:** Darshan Shimoga Chandrashekar, Poulami Dey, Kshitish K. Acharya

**Affiliations:** 1 Institute of Bioinformatics and Applied Biotechnology (IBAB), Biotech Park, Electronic City, Bengaluru (Bangalore), 560100, Karnataka state, India; 2 Manipal University, Manipal, 576104, Karnataka state, India; 3 Shodhaka Life Sciences Pvt. Ltd., IBAB, Biotech Park, Bengaluru (Bangalore), 560100, Karnataka state, India; University of Lausanne, SWITZERLAND

## Abstract

**Background:**

Genome-wide repeat sequences, such as LINEs, SINEs and LTRs share a considerable part of the mammalian nuclear genomes. These repeat elements seem to be important for multiple functions including the regulation of transcription initiation, alternative splicing and DNA methylation. But it is not possible to study all repeats and, hence, it would help to short-list before exploring their potential functional significance via experimental studies and/or detailed *in silico* analyses.

**Result:**

We developed the ‘Genomic Repeat Element Analyzer for Mammals’ (GREAM) for analysis, screening and selection of potentially important mammalian genomic repeats. This web-server offers many novel utilities. For example, this is the only tool that can reveal a categorized list of specific types of transposons, retro-transposons and other genome-wide repetitive elements that are statistically over-/under-represented in regions around a set of genes, such as those expressed differentially in a disease condition. The output displays the position and frequency of identified elements within the specified regions. In addition, GREAM offers two other types of analyses of genomic repeat sequences: a) enrichment within chromosomal region(s) of interest, and b) comparative distribution across the neighborhood of orthologous genes. GREAM successfully short-listed a repeat element (MER20) known to contain functional motifs. In other case studies, we could use GREAM to short-list repetitive elements in the azoospermia factor a (AZFa) region of the human Y chromosome and those around the genes associated with rat liver injury. GREAM could also identify five over-represented repeats around some of the human and mouse transcription factor coding genes that had conserved expression patterns across the two species.

**Conclusion:**

GREAM has been developed to provide an impetus to research on the role of repetitive sequences in mammalian genomes by offering easy selection of more interesting repeats in various contexts/regions. GREAM is freely available at http://resource.ibab.ac.in/GREAM/.

## Introduction

Repetitive sequences are known to occupy significant portions of the genomes of many complex multicellular organisms [1]. Following earlier indications that all such repeats may not be just ‘junk’ DNA [2–6] researchers have been paying increased attention to these genomic repeat sequences in various contexts, which include gene expression regulation, as described below.

Barbara McClintock indicated the role of 'transposable elements' in ‘genic expression' very early [7, 8]. Involvement of genomic repeat elements in the transcription regulation via defined chromatin loops was suggested based on the high density of transposons in scaffold/matrix associated regions [9]. Multiple experimental evidences for the role of many repeat elements in the regulation of gene expression have accumulated in the recent years. In fact, genomic repeat elements are now indicated to have a significant role in the transcriptional control and/or regulatory networks. For example, Lynch et al [10] found the DNA transposon MER20 to be enriched in the neighborhood of differentially regulated genes in the endometrial stroma and provided evidence for the transcription factor binding abilities of DNA-motifs present within this repeat element. The density of repeat elements and/or their methylation levels have been suggested to influence the regulation of expression of neighboring genes [11–13]. Kunarso et al [14] indicated significant contribution of transposable elements to the occurrence of some of the transcription factor (OCT4 and NANOG) binding sites. They suggested that transposable elements have incorporated new genes into the core regulatory network of embryonic stem cells in humans and mice, and that species-specific transposable elements have substantially altered the transcriptional circuitry of pluripotent stem cells. Lowe et al [15] also suggested a role for certain mobile elements in shaping the gene regulatory networks across mammalian genomes. This suggestion was made because such elements contributed to at least 5.5% of the mammal-exclusive nonexonic conserved elements located in the gene deserts with a strong preference for the neighborhood of genes involved in the development and transcriptional regulation. The MER121 repeat class seems to be conserved in the orthologous genomic locations and may play a cis-regulatory or structural role in mammalian genomes [16]. Genomic repeats have been specifically implicated in stress-responses of plants via gene expression regulation [17, 18]. However, more research efforts are needed to investigate the possible role of the repeat elements in transcriptional regulation and regulatory networks in different species, tissues and conditions.

Genomic repeat elements seem to be important from other perspectives such as evolution, genomic stability, alternative splicing and pathogenicity [19–34]. For example, studying repetitive sequences/elements may help to understand the organization and evolution of eukaryotic genomes [19–22]. Repeat elements have been reported to be associated with some types of cancer [23–24]. Interspersed repeat elements at 'breakpoints' may be relevant to specific reciprocal translocations, which in turn may be central to the pathogenesis of chronic myeloid leukemia [25]. Insertion of the repeat elements have caused in new exons from introns (exonization) or introns from exons (intronization) [26–27] and thus contributed to diversity in terms of alternative splicing in mammals [28–29]. Differential methylation patterns associated with the repeat elements within introns may be associated with the alternative splicing, and seed coat colors in soybean lines [30]. Zabala et al [31] suggested radiation induced temporal changes in methylation within some of the repeat elements in the mouse genome. This study also noted the dependence of such changes on the genetic background (type of strain), gender and the type of the repeat elements.

Thus, apart from the need to explore genomic repeat elements in the context of transcriptional regulation, there is a need to study them in the context of other cellular/molecular processes in mammalian species. But, at present it would be extremely difficult, or even impossible to explore every repeat element present in specific regions such as the neighborhood of a set of genes with interesting and shared expression patterns.

In this context, it is necessary to screen all genomic repeat elements and short-list the interesting ones, which may have a higher chance of being functionally relevant. For instance, it would benefit researchers to select a few repeat elements such as those enriched in the regions around the transcription start sites of a set of differentially transcribed genes. It would then be feasible to explore the types of TFBSs and their abundance within each selected repeat element, hypothesize about their possible functions and/or experimentally test such functional roles. Analyzing the distribution of repeat elements conserved around a set of the orthologous genes can be another type of study where a preliminary short-listing can help. Similarly, it would be interesting to study the repetitive element(s) enriched in two or more species in the neighborhood of genes with constitutive expression in all species considered [see the utility section below for more examples].

There has been a need for bioinformatics support to analyze a variety of possible functions of the repeat elements [35]. Different tools have already been developed for detection and masking analyses of the repeat elements [36]. A comparison of these tools [37] reveals that none of them allows identification of over-/under-represented repeat elements in the neighborhood of a given set of genes. TranspoGene [38] allows querying with multiple gene names and reveals the repeat elements from intronic, exonic and proximal promoter (up to 250 base pairs upstream) regions of the genes. But even this tool cannot be used to analyze the repeat element distribution across genes. Thus, there is a requirement to short-list the repeats with a statistically significant difference in their distribution within the regions of interest, compared with the distribution in the whole genome.

Hence, a new online computational tool, ‘Genomic Repeat Element Analyzer for Mammals’ (GREAM, http://resource.ibab.ac.in/GREAM) has been developed to identify over-/under-represented elements in, a) the promoters or other neighborhoods across a set of query genes from a species, b) specific chromosomal regions of a species, and c) the promoters or other neighborhoods of orthologous genes from different species.

## Materials and Methods

### Data collection

RepeatMasker [http://www.repeatmasker.org/] based repeat annotation tracks of the mammalian genome sequences were downloaded from NCBI Genome FTP site (ftp://ftp.ncbi.nlm.nih.gov/genomes/MapView/). Similarly, gene-related information for all 17 mammalian species was obtained from NCBI Gene FTP site (ftp://ftp.ncbi.nih.gov/gene/). Gene identifiers (official gene symbol, Entrez Gene id, Ensembl Gene id, RefSeq mRNA id and UniGene id) corresponding to all mammalian species considered were downloaded from Ensembl BioMart (http://asia.ensembl.org/biomart/martview). Orthologous gene information was downloaded from NCBI HomoloGene Build 67 (ftp://ftp.ncbi.nih.gov/pub/HomoloGene/). Additional information about the repetitive elements was downloaded from Repbase 19.07 (http://www.girinst.org/repbase/). The gene ontology (GO) details and GO-gene association files were downloaded from Gene Ontology Consortium (http://geneontology.org/page/download-ontology, http://geneontology.org/page/download-annotations).

### Existing software and programming languages used to create GREAM

Apache 2.2 [http://httpd.apache.org/] was used as a web server on a 64-bit machine with 32 GB RAM and 3 TB HDD space, having Red hat Linux as operating system. MySQL RDBMS [MySQL 5.1.69, http://www.mysql.com/] was used for the data storage. GUI was developed using PERL-CGI scripts. Web technologies such as Cascade Style Sheets (CSS) and JavaScript (JSS) were used for implementing user-friendly features. PHP 5.3.3 was used to create a graphical representation of repetitive element distribution in the neighborhood of all query genes. Open flash chart [http://teethgrinder.co.uk/open-flash-chart/] was used to create the histograms representing relative abundance of the repetitive element classes within the gene neighborhoods.

### Obtaining genome-wide frequency of occurrence for every repetitive element based on two types of statistical analyses

The genome-wide statistics for the frequency of occurrence of each repeat element was first calculated in two ways: a) by considering the fraction of the genes that have the specific repeat element in their neighborhood (gene counts); b) by considering the fraction of the specific repeat element among all repeats in the genome (repeat counts).

Genome-wide statistics for a repeat element based on the 'gene counts' is the ratio of the 'number of genes, each of which has that specific repeat element at least once in its neighborhood' to 'all genes'. This ratio can be represented by the following formula:

$$\frac{Count\;of\;genes\;with\;the\;specific\;repeat\;in\;their\;neighborhood}{Total\;number\;of\;genes\;in\;the\;genome}$$

Genome-wide frequency of a specific repeat element based on ‘repeat counts’ is the ratio of 'occurrence of that repeat element' to the 'occurrence of all repeat elements'—in specific gene neighborhoods. This ratio can be represented by the following formula:

$$\frac{Count\;of\;the\;specific\;repeat\;element\;around\;the\;genes}{Count\;of\;all\;repeat\;elements\;around\;the\;genes}$$

According to the above-mentioned formulae, PERL scripts were written to calculate the genome-wide frequency of occurrences for all repetitive elements—for each type and size of the gene neighborhoods. The types of gene neighborhood included upstream transcription start site (TSS), downstream of TSS, downstream of gene end, on both sides of the TSS [around TSS] or before and after the gene [around the gene]. In genes known to code for multiple, alternatively spliced transcripts, the 5’-most TSS have been picked. Downstream of the TSS may include one or both UTRs and one or more exons as well as introns. The different sizes considered around the genes or the TSS were 10 kb, 20 kb, 50 kb, 100 kb, 200 kb, 500 kb, 1 mb, 1.5 mb, 2 mb, 2.5 mb and 3 mb. These pre-computed genome-wide frequencies of the repeat elements will be compared with the observed frequency from around the genes in a query set and then its difference with the corresponding genome-wide frequency would be computed. Codes have been written to compute the significance of the difference as described below.

### Estimating of over-/under-representation of repetitive elements

GREAM estimates the observed frequency of a specific repeat element for a given gene-set, based on the ‘gene counts’ as,

$$\frac{Count\;of\;query\;genes\;with\;the\;specific\;repeat\;in\;their\;neighborhood}{Total\;number\;of\;query\;genes}$$

OR based on ‘repeat counts’ as,

$$\frac{Count\;of\;the\;specific\;repeat\;in\;the\;neighborhood\;of\;all\;query\;genes}{Count\;of\;all\;repeats\;in\;the\;neighborhood\;of\;all\;query\;genes}$$

For both types of analyses, the ratio of the observed frequency of occurrence of each repetitive element in the query region to the corresponding genome-wide frequency would be determined. This ratio indicates the over-/under-representation of each repeat. The significance of the ratio (i.e., the difference between the two frequencies) would be assessed by the binomial probability [39] for each repeat element, using the following formula:

$$P(X=k)=\left(\begin{array}{l} n\\ k \end{array}\right)\;p^{k}\;{\left(1-p\right)}^{n-k}$$

In case of the analysis using the ‘gene counts’, ‘p’ is the genome-wide frequency of occurrence (described in the previous section) of a specific repeat element, ‘n’ is the total number of query genes submitted and 'k' is the total number of query genes having the repeat element at least once in their neighborhood.

In case of the analysis by using the ‘repeat counts’, ‘p’ is the genome-wide frequency of occurrence (described in the previous section) of a specific repeat element, ‘n’ is the total occurrence of all repetitive elements in the neighborhood of all query genes (for ‘Analyze gene-set’ and ‘Analyze orthologous gene-set’ feature) or in the specified chromosomal location (for ‘Analyze locus’ feature), and 'k' is the total occurrence of a specific repeat element in the neighborhood of all query genes (for ‘Analyze gene-set’ and ‘Analyze orthologous gene-set’ feature) or in a specific chromosomal location (for ‘Analyze locus’ feature).

If the calculated probability value (P) is less than 0.05, over-/under-representation of specific repetitive element was considered to be significant.

### Estimation of repeat element coverage on mammalian genomes

Genome size for human and mouse was obtained from the genome reference consortium [http://www.ncbi.nlm.nih.gov/projects/genome/assembly/grc/], while for other species the information was obtained from Ensembl genome browser [40]. The size of the genome covered by repeat elements was computed by parsing the repeat annotation tracks of specific genome assemblies (downloaded from NCBI Genome ftp).

### Transcription factor binding site (TFBS) analysis of repetitive element sequences

Sequences of the repetitive elements obtained from Repbase 19.07 were screened for potential TFBSs using the CLOVER tool (http://zlab.bu.edu/clover/) [41]. Position weight matrices of TFBSs are downloaded from JASPAR 2014 (http://jaspardev.genereg.net/) [42]. The locations of potential TFBSs were listed using CLOVER (with default threshold score).

### Case study for validation

Lynch et al [10] reported association of a DNA transposon, MER20 in rewiring the regulatory network of differentially regulated stromal genes. They reported enrichment of MER20 within 200KB of each end of differentially expressed genes, identified TFBSs within MER20 (using TRANSFAC database), and confirmed their transcription factor binding ability via ChIP-qPCR experiments. Hence, MER20 formed a good case for validating the efficiency of GREAM; it is perhaps the only available repeat with such evidences. We downloaded processed RNA-seq data for differentiated and undifferentiated human endometrial stromal cells from GEO (GSE30708), obtained 1,149 differentially expressed genes having a read count of >20 in either differentiated or undifferentiated endometrial stromal cells as mentioned in the original study, and analyzed the repeat element distribution in the same neighborhood region (around gene, 200KB). Repeat enrichment was analyzed using statistics based on ‘gene counts’.

## Results and Discussion

The distribution of repeat elements in the mammalian genomes was first reviewed by quantifying them in the latest genome-builds available (Table 1). 'Annotated repeat elements' constitute 34 to 55% of known mammalian genomes. Non-mammalian genomes seem to have significantly lesser proportion of such repeats [43]. But there can be exceptions. For example, repeat elements contribute to 77% of the genome in *Rana sculenta* [1, 44]. In general, the distributions noted by us were similar to the observation made earlier by other researchers for human and mouse species [1, 44]. The estimations may vary to some extent based on the method used. Compared to the conventional method (Repeat Masker, which involves aligning the repeat consensus sequences with the genome) of repeat element identification, a novel *de novo* strategy (P-cloud) estimated higher (i.e. 69%) repeat element coverage of the human genome [45].

**Table 1:** The contribution of repeat elements to nuclear genomes in 17 mammalian species.

Species	Genome assembly	Total size (in nucleotides)	Repeat coverage (in nucleotides)	Percentage of genome covered by repeat elements
Human	GRCh37.p5-Primary Assembly	3,101,788,170	1,441,122,130	46.46
Mouse	GRCm38-C57BL/6J	2,730,855,475	1,186,206,269	43.44
Rat	Rnor_5.0-Primary Assembly	2,573,362,844	1,103,002,536	42.86
Cow	Btau_4.6.1-Primary Assembly	2,649,685,036	1,347,191,424	50.84
Chimpanzee	Pan_troglodytes-2.1.4-Primary Assembly	2,995,917,117	1,458,594,710	48.69
Rabbit	OryCun2.0-Primary Assembly	2,604,023,284	1,115,709,440	42.85
Dog	CanFam3.1-Primary Assembly	2,392,715,236	1,004,202,711	41.97
Elephant	Loxafr3.0-Primary Assembly	3,118,565,340	1,456,307,490	46.70
Gibbon	Nleu_3.0-Primary Assembly	2,756,591,777	1,392,833,579	50.53
Gorilla	gorGor3.1-Primary Assembly	2,828,888,833	1,344,286,159	47.52
Horse	EquCab2.0-Primary Assembly	2,428,790,173	1,046,969,418	43.11
Monkey	Mmul_051212-Primary Assembly	3,093,871,206	1,466,974,355	47.42
Marmoset	Callithrix jacchus-3.2-Primary Assembly	2,759,289,125	1,301,711,202	47.18
Opposum	MonDom5-Primary Assembly	3,501,660,299	1,951,703,622	55.74
Orangutan	P_pygmaeus_2.0.2-Primary Assembly	3,109,347,532	1,563,466,038	50.28
Pig	Sscrofa10.2-Primary Assembly	3,024,658,544	1,040,672,576	34.41
Platypus	Ornithorhynchus_anatinus-5.0.1-Primary Assembly	1,917,748,604	889,518,843	46.38

### Overview of GREAM

**Fig 1:**
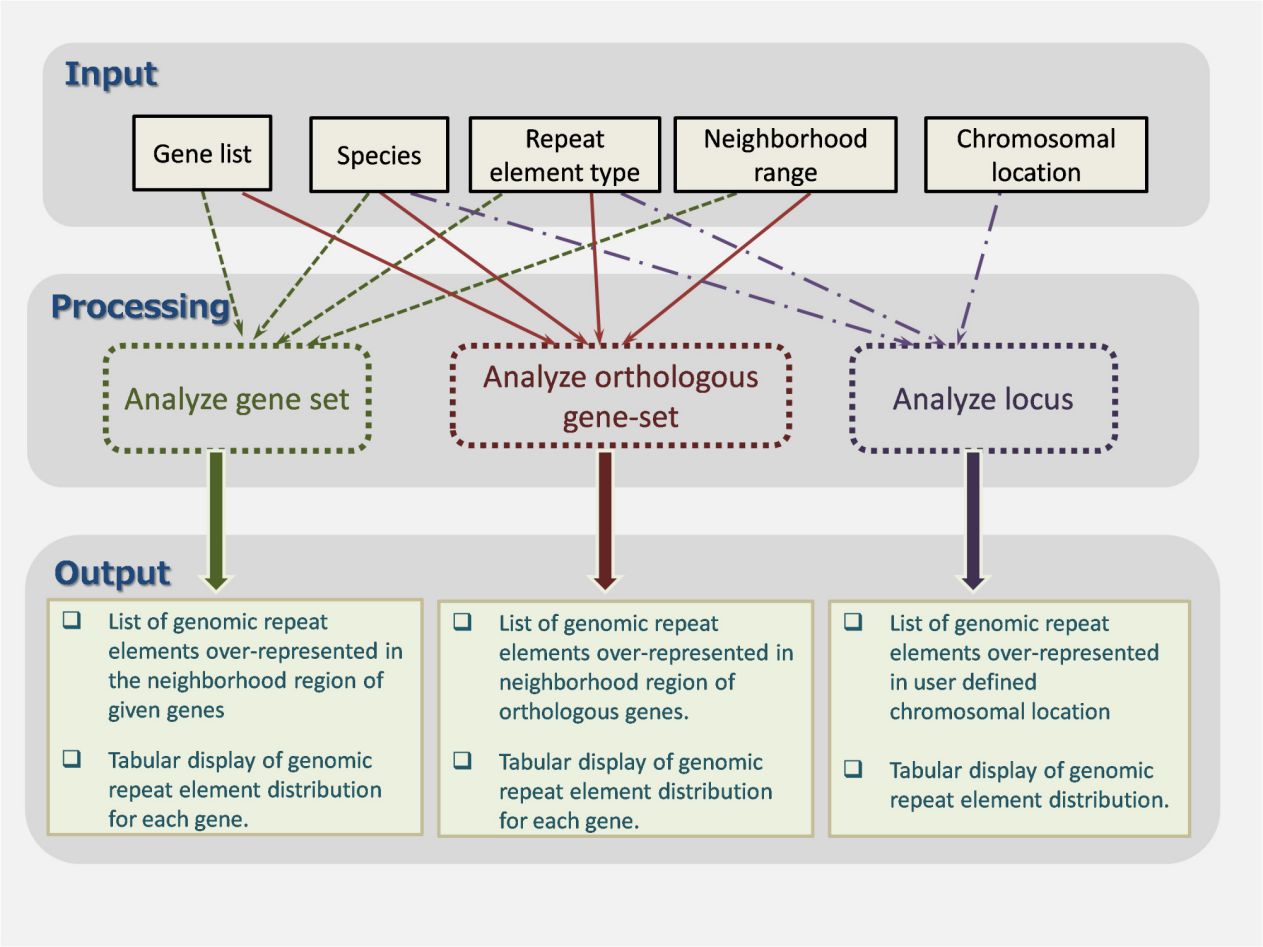
Workflow of the GREAM web server.

In Fig 1, the overall workflow of GREAM is schematically represented.

GREAM allows three types of repeat element analysis: 1) the feature ‘Analyze gene-set’ facilitates analysis of the repeat element distribution within the neighborhood of a query gene-set; 2) the feature ‘Analyze orthologous gene-set’ facilitates analysis of the repeat element distribution in the neighborhood of a query gene-set and their orthologous genes; 3) the feature ‘Analyze locus’ facilitates analysis of the repeat element distribution within specific chromosomal location(s).

#### Input data

GREAM requires a set of genes as an input for both ‘Analyze gene-set’ and ‘Analyze orthologous gene-set’ features. User can either paste the gene identifiers in the text area provided or upload the gene list as a text file. Official gene symbols, Entrez gene identifiers, Ensembl gene identifiers, RefSeq mRNA identifiers or the Unigene identifiers can be used to upload the gene-list. But the submitted query list should be homogeneous. The species can be chosen from the options provided in a drop down menu. ‘Analyze gene-set’ can be used for 17 mammalian species. ‘Analyze locus’ can be used for all species except elephants and gibbons as the contigs were not assembled into chromosomes for these two species. ‘Analyze orthologous gene-set’ can be used for only seven species (human, mouse, rat, cow, dog, chimpanzee and monkey) as HomoloGene includes orthologous gene information exclusively for these mammalian species.

The tool allows user to specify the gene neighborhood and the population statistics to be considered (‘gene counts’ or ‘repeat counts’) for short-listing important repeat elements. In case of ‘Analyze orthologous gene-set’, additional species need to be selected. For ‘Analyze locus’ feature, GREAM requires specification of a) a chromosomal location (for example, Chr1:3011498–3120000 or Chr4|NT_113885.1:5000–100000 or ChrUn|NT_167210.1:100–150000), b) mammalian species and c) type of genomic repeat element of interest (‘all’ repeats scanned with default settings).

There is an option within each of the three types of analyses for users to provide a title for the job submitted to keep track of their own analysis, and also to use e-mail address for receiving a link for the output on completion of submitted job.

#### Job handling

Each submitted job will be stored and processed based on the order of submission using a PERL script that runs on the server. User can monitor the submitted job’s status (which can be either “in queue”, “being processed” or “completed”) through the ‘job status’ page. On completion of the job, the user will be redirected to the result page. In addition, a link to the result page will be sent to the e-mail address–if provided. The result of the analysis of a specific job can be accessed till one week of its submission date.

#### Processing input

In ‘Analyze gene-set’, the query genes are first subjected to gene identifier conversions, using a local copy of the Ensembl Biomart, to obtain corresponding NCBI Entrez gene identifiers. Their chromosomal locations are then obtained using the gene annotation track from NCBI Gene and based on the input specifications, the neighborhood to be analyzed are obtained for all query genes. Using the repeat annotation tracks downloaded from NCBI, the distribution of all or user predefined repeat elements within the neighborhood regions of all query genes will be obtained. Observed frequency of each repeat element is compared with its pre-computed genome-wide frequency, for the user-specified statistical parameters (‘gene counts’ or ‘repeat counts’).

In ‘Analyze locus’, the user predefined chromosomal location will be scanned for the presence of all or selected repetitive elements using repeat annotation tracks downloaded from NCBI. Then, the observed frequency of occurrence of each repeat element in the given region will be compared with their genome-wide frequency of occurrence within the entire genome. The analysis is based on ‘repeat counts’ statistics.

In ‘Analyze orthologous gene-set’, the gene identifier conversion of query genes will be performed as mentioned before. A local copy of the ‘HomoloGene’ database will then be used for obtaining orthologous genes corresponding to the query gene-set and the selected mammalian species. Gene neighborhood of query gene-set and each of orthologous gene-sets will be scanned separately for all or user predefined repetitive elements. The observed frequency for the query-set will be compared with the genome-wide frequency of occurrence of each repeat element, for each species as per user-specified statistics (‘based on gene counts’ or ‘based on repeat counts’). Finally, the repeat elements that show significant over-/under-representation in the query gene-set and its orthologous gene-sets from at least one or other species are identified.

In all cases, the binomial probability (see methods for more detail) will be used to calculate the significance of difference of the frequency in the query regions versus the comparable genome-wide frequency of occurrence of each repeat element.

#### Output

‘Analyze gene-set’ feature results in a table of the repeat elements and their location within neighborhood and a flash chart showing the percentage of detected repeat elements of different types for each queried gene. Also a summary table will be displayed with details of the repeat elements that are statistically over-/under-represented within the specified size and type of neighborhood. GREAM allows users to visualize the distribution of one or more significant repeat elements within the regions of interest. This feature is available when a user chooses one of the following as the type of neighborhood region: ‘around TSS’, ‘upstream of TSS’, ‘downstream of TSS’ or ‘downstream of gene end’.

In ‘Analyze locus’ feature, the output page includes a table for the repeat elements and their location within the queried chromosomal location and a flash chart showing percentage of the detected repeat elements of different types. A summary table provides details of the repeat elements that are statistically over-/under-represented within the given chromosomal location.

In ‘Analyze orthologous gene-set’ feature, similar results are provided for all queried genes and their orthologous counterparts of chosen mammalian species: a) a table of the repeat elements and their location within the neighborhood and b) a flash chart showing percentage of the detected repeat elements of different types. Summary tables reveal the details of the repeat elements that are statistically over-/under-represented within specific regions around the query genes and the corresponding orthologous genes.

Each repeat element represented in tables will be hyperlinked to a web page showing further details—including the sequence and potential transcription factor binding sites within the sequence. GREAM facilitates ontology analysis of genes that have enriched repeat elements in their neighborhood.

### Validating GREAM using a case study

Lynch et al [10] showed that MER20, a DNA transposon is enriched within a 200kb neighborhood of endometrially expressed genes. They suggested the involvement of MER20 in co-ordinating gene expression regulation via the TFBSs harbored within it. By using ‘Analyze gene-set’ feature of GREAM, we investigated the neighborhood of 1,149 differentially expressed genes obtained from their study. The goal was to find out if GREAM can identify one of the repeat elements detected independently by another group of researchers, and also known to harbor TFBSs with proven TF-binding capacities. GREAM indeed found MER20 to be significantly (p-value: 0.0234) over-represented in the neighborhood of 943 (82%) of the query genes.

It is important to note two aspects of GREAM’s validation: a) the tool short-listed a repeat element that was independently identified earlier using a different statistical approach (Yates corrected chi-square test used by Lynch et al [10], as against the ‘binomial probability of repeat counts’ employed here); b) the detected repeat element was already shown to have TFBSs with functional evidences via ChIP-qPCR [10]

GREAM could detect other potentially important repeats. In fact, 21 repeat elements were over-represented with p-value < = 0.0234, in the regions studied, including L1MB7, MIR3, L1ME4a, L1MC4 and MLT1D with p-value 0.0001 or less (see S1 Table for more details). Our results indicated the probability of a larger number of repeat elements in addition to MER20, having a functional role in regulating transcription of the pregnancy-related genes investigated originally [10] and selected for the analysis in our study. But it should be noted that there could be false positives among the repeats short-listed by the tool. Further experimental investigations are needed to confirm or reject the relevance of genomic repeats indicated by GREAM.

### Utility of GREAM

The above-mentioned case study illustrates the use of GREAM for short-listing the genomic repeat elements around genes that may have a functional significance. Thus, the tool can be used to short-list important repeat elements for detailed *in silico* analysis/experimentation. The following questions represent more examples of research problems that can be addressed using GREAM:

1. Are there repetitive elements associated with the epigenetic regulation of promoters of certain genes sharing functionalities or expression pattern?
2. Is there an abundance of any specific type of repeat element in the neighborhood of chromosomal regions that are frequently associated with genetic recombination or a specific type of structural abnormality?
3. Are there X- or Y-chromosome-associated repeat elements preserved across mammalian species and, if yes, in which regions?
4. Are there specific type of repeat elements around genes that are near telomeres and/or centromeres?

We describe three case studies for a better illustration of the utilities (the repeat elements detected for each of these case studies are listed in supporting information:

### A. Analyzing the repeat element distribution in the neighborhood of rat genes associated with general liver injury

**Fig 2:**
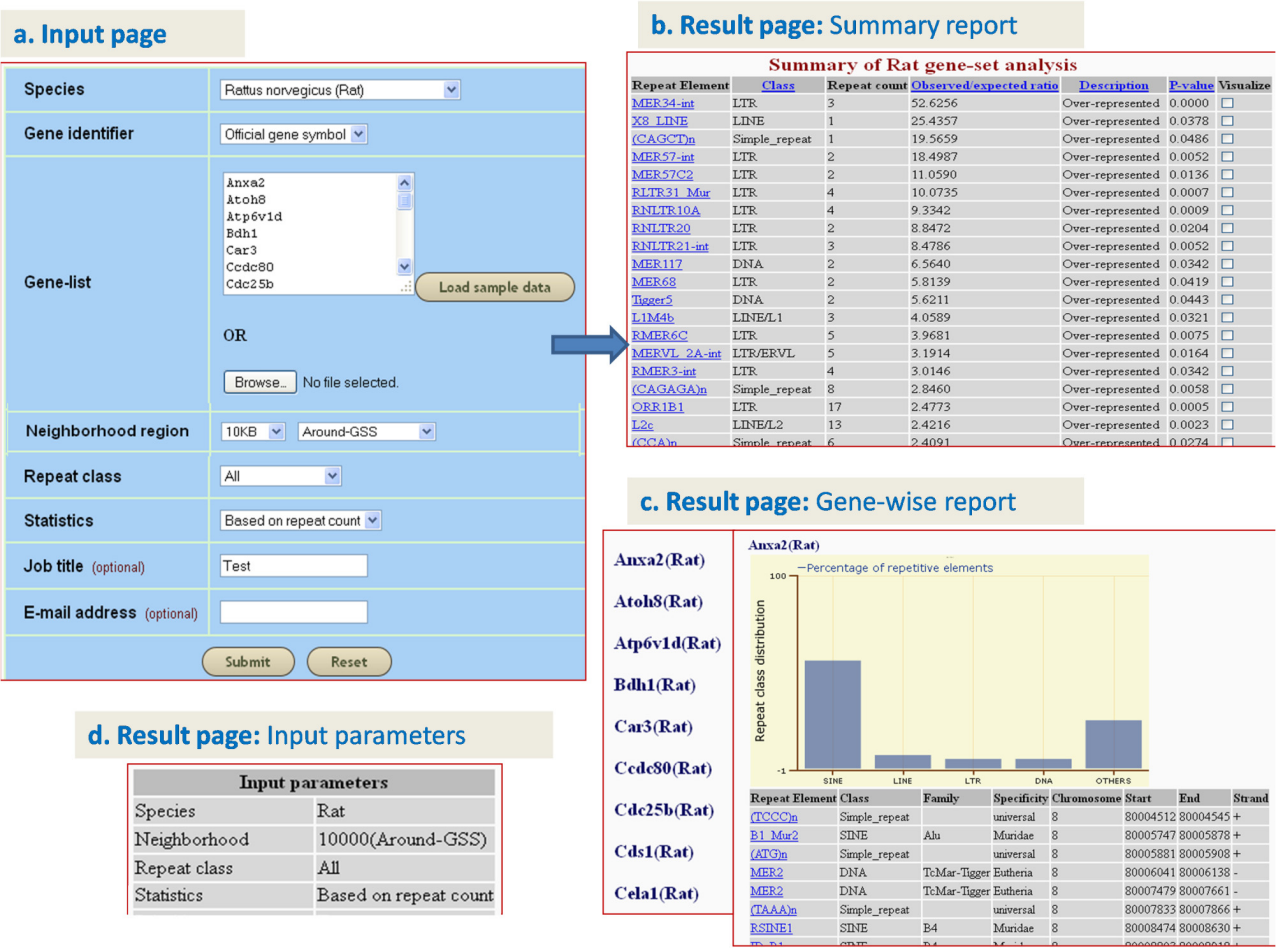
Snapshots of GREAM illustrating use of ‘analyze gene-set’ option on a gene-set from Tawa et al [46].

‘Analyze gene-set’ option was used to identify over-represented repeat elements in the neighborhood of 64 rat genes reported to be associated with the liver injury based on gene expression profile [**46**]. Input parameters such as species (rat), gene identifier (official gene symbol), neighborhood region (10 kb nt, around TSS), repeat class (all), statistics (based on ‘repeat counts’) and job name (‘rat-liver-injury-set’) were provided in addition to the list of 64 genes [Fig **2**A]. On submission, the job was scheduled for the processing, and after processing the result page revealed the following: 1) ‘Summary report’ table with a list of 81 significant repeat elements (65 over-represented and 16 under-represented) from the neighborhood of the 64 query genes. The significance of many of the over-represented repeats was remarkable with very low p-values. For example, the top five repeats were over-represented with a p-value of less than 10^−4^ and included three LTR retrotransposons (ERVB1_2-LTR_RN, MER34-int, RLTR18-int) two DNA transposons (Zaphod2, Ricksha_c). On the other hand, even the top five under-represented repeats (LINEs: Lx, Lx5, Lx8b, L1_Rn and L1_Rn2) had only a moderate p-value (p<0.021) (S**4**and S**5**Tables). When the analysis was repeated with the statistics option of ‘gene counts’, 45 over-represented and 3 under-represented repeat elements were found, with moderate p-values. While the three under-represented repeat elements were LINEs (Lx, Lx8b and Lx5) (p<0.003), the top five (p< = 0.006) over-represented repeat elements included LTR retrotransposons (RLTR31_Mur, MTEa and MER67B), a SINE element (ID4_) and a LINE element (L2c). Of these 48 elements, 43 were the same as those obtained with ‘repeat counts’ option (S**6**and S**7**Tables). The table showed observed/expected ratio of repeat elements, corresponding p-values and count of each repeat type [Fig **2**B] 2) ‘Gene-wise report’, with an account of the repeat element distribution in the neighborhood of each of 64 genes submitted in the form of histogram and table [Fig **2**C]. The results page also display the ‘Input parameters’ provided initially [Fig **2**D].

### B. Analyzing the repeat element distribution in AZFa region of human Y chromosome

**Fig 3:**
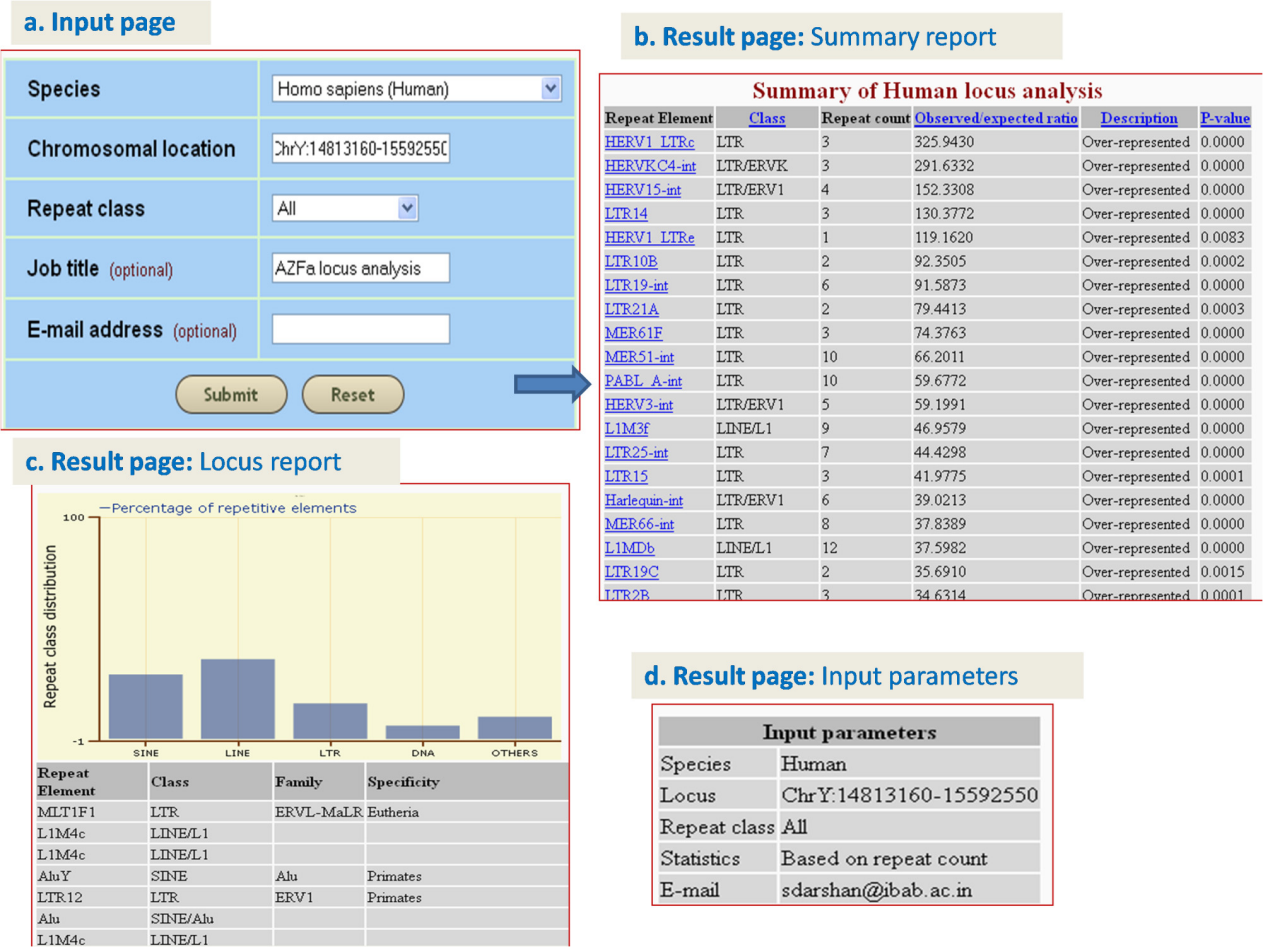
Snapshots of GREAM illustrating use of ‘analyze locus’ option on AZFa region.

‘Analyze locus’ option was used to identify the repeat elements that are enriched in the AZFa locus region of human Y chromosome. This locus, which has 3 genes (USP9Y, DBY and UTY) that directly influence male fertility [47] was submitted as a query (chromosome Y: 14813160 to 15592550). Additional parameters such as species (human), repeat class (All) and job name (‘AZFa locus analysis’) were specified [Fig 3A]. ‘Summary report’ table was displayed listing 111 repeat elements (91 over-represented and 20 under-represented) in the region of interest along with the counts of repeat elements, their observed/expected ratios and corresponding P-values [Fig 3B]. ‘Locus report’ provided distribution of the repeat elements in AZFa region in the form of histogram and table [Fig 3C], and ‘Input parameters’ were also displayed [Fig 3D]. The results included 35 over-represented repeats with significant p-value (p<10^−4^). Examples include LTR retrotransposons (HERV1_LTRc, HERVKC4-int, HERV15-int, LTR14, LTR19-int), LINEs (L1M3f, L1MDb, L1M3c, L1M4c, L1MB4, L1MCa) and DNA transposons (Tigger4, Tigger7, Tigger2). Similarly, the under-represented elements included two LINEs (L2a and L2c) and three SINEs (MIR, MIRb and MIR3) with p<10^−4^ (S**2**and S**3**Tables).

### C. Analyzing the repeat element distribution in the neighborhood of genes coding for transcription factors and showing conserved expression patterns across human and mouse

**Fig 4:**
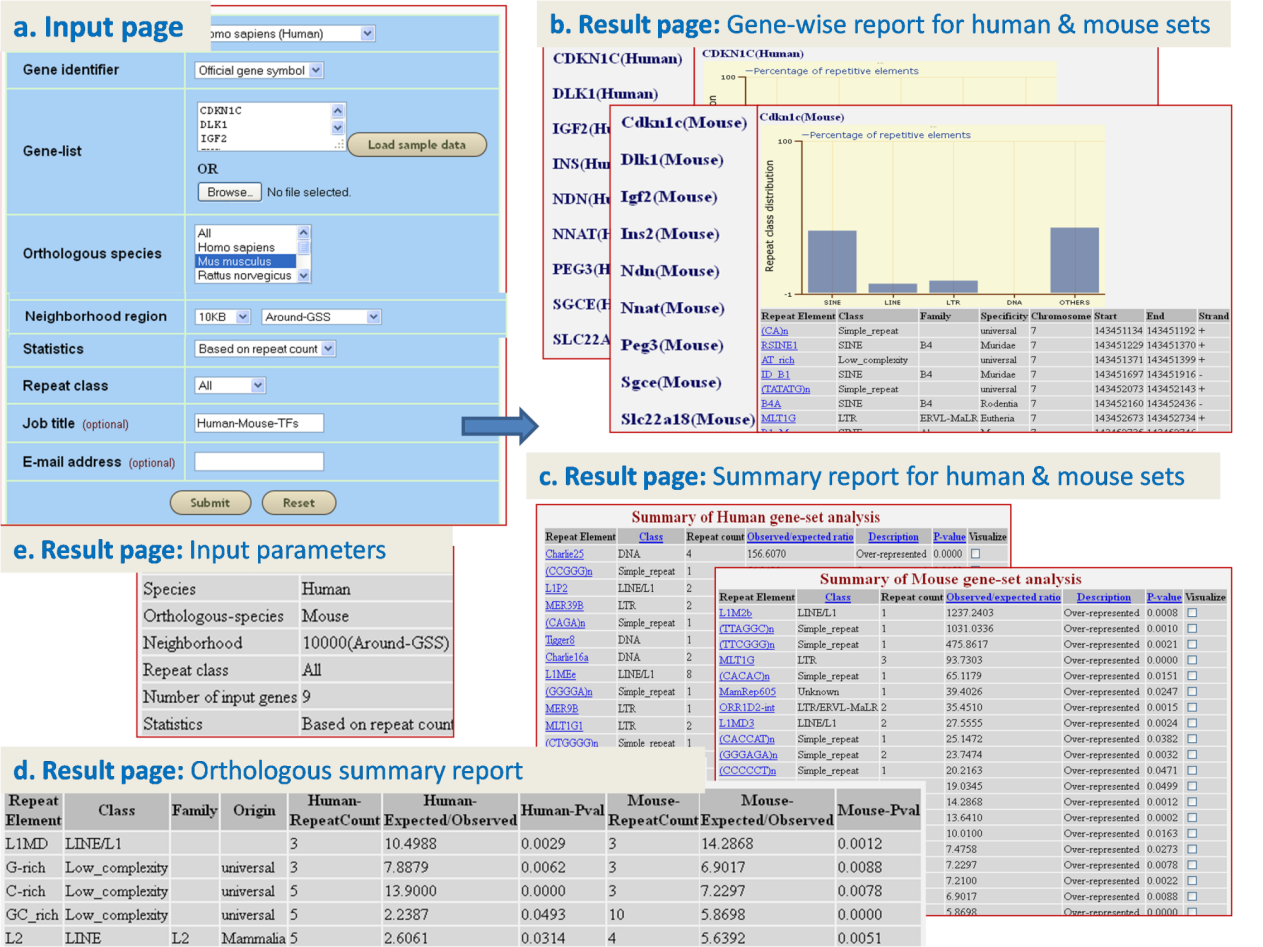
Snapshots of GREAM illustrating use of ‘analyze orthologous gene-set’ on human transcription factor genes and their mouse orthologs from Steinhoff et al [48].

Steinhoff et al [48], found that the levels of 9 transcription factor-coding mRNAs (CDKN1C, DLK1, IGF2, INS, NDN, NNAT, PEG3, SGCE and SLC22A18) had a positive correlation across 22 human and mouse tissues. We used ‘analyze orthologous gene-set’ feature of GREAM to identify the repeat elements enriched in the neighborhood of the orthologs of interest. The selected 9 genes were input along with other parameters such as species (human), gene identifier (gene symbol), orthologous species (mouse), neighborhood region (10 kb nt, around TSS), statistics (based on ‘repeat counts’), repeat class (all) and job name (‘Human-Mouse-TFs’) [Fig 4A]. The ‘Summary report’ table for the human gene-set listed 24 over-represented repeat elements and 3 under-represented ones, while the table for mouse orthologs listed 25 over-represented repeat elements and 3 under-represented [Fig 4B]. ‘Gene-wise report’ for human and mouse gene-set showed distribution of repeat elements in neighborhood of individual genes in the form of histogram and table [Fig 4C]. ‘Orthologous summary report’ listed 5 repeat elements (L1MD, G-rich, C-rich, GC_rich and L2) commonly enriched in the human gene-set and their mouse ortholog gene-set [Fig 4D], and 5) ‘Input parameters’, were also displayed [Fig 4E]. Result details are available at S8, S9, S12, S13 and S16 Tables. When the analysis was repeated with the statistics option of ‘gene counts’, for the human gene-set, 13 over-represented and 5 under-represented repeat elements were found. Of these 18 repeats, 16 were the same as those obtained using the ‘repeat counts’ option. In case of mouse ortholog gene-set, 13 over-represented and 4 under-represented repeat elements were found. Of these 17 repeats, 16 were the same as those obtained using the ‘repeat counts’ option for mouse. G-rich and C-rich elements were found to be enriched in the human gene-set and their mouse ortholog gene-set. These two were found to be common among human and mouse gene-sets when ‘repeat counts’ option was used. Result details are available at S10, S11, S14, S15 and S17 Tables.

### Limitations

While short-listing the statistically over-/under-represented repeat elements is one way of finding potentially functional ones, many such short-listed elements may not be really associated with functions. But, in absence of alternatives, this approach can assist scientists in exploring repetitive elements. Considering short-listed repeats with stringent p-value cut offs such as p<0.005, may reduce the number of false positives obtained from GREAM.

The time taken by GREAM for processing the jobs submitted is directly proportional to the size of the gene-set, neighborhood regions considered and type of statistics used. Analysis using the ‘repeat counts’ option usually takes longer time than ‘gene counts’ option. Analyses with larger regions (1 mb and above) can take a few minutes to hours, depending on the number of genes (please see user guide of the tool for more details).

## Conclusions

In the context of exploring multiple possible functional roles, GREAM would be useful for selecting important genomic repeat elements present around specific genes or in chromosomal regions of interest. With the help of this web server, many researchers can now raise new questions and/or progress faster in their investigation of the potential functions of repetitive sequences.

## Supporting Information

S1 Table Summary of repeat element analysis on human differentially regulated genes of endometrial stromal cells. (DOCX)

S2 Table Summary of over-represented repeat elements found within AZFa locus of the human Y chromosome which influence male fertility. (DOCX)

S3 Table Summary of under-represented repeat elements found within AZFa locus of the human Y chromosome which influence male fertility. (DOCX)

S4 Table Summary of repeat elements, over-represented (based on ‘repeat counts’) in the neighborhood of 64 rat genes associated with general rat injury. (DOCX)

S5 Table Summary of repeat elements, under-represented (based on ‘repeat counts’) in the neighborhood of 64 rat genes associated with general rat injury. (DOCX)

S6 Table Summary of repeat elements, over-represented (based on ‘gene counts’) in the neighborhood of 64 rat genes associated with general rat injury. (DOCX)

S7 Table Summary of repeat elements, under-represented (based on ‘gene counts’) in the neighborhood of 64 rat genes associated with general rat injury. (DOCX)

S8 Table Summary of repeat elements, over-represented (based on ‘repeat counts’) in the neighborhood of 9 human transcription factor genes. (DOCX)

S9 Table Summary of repeat elements, under-represented (based on ‘repeat counts’) in the neighborhood of 9 human transcription factor genes. (DOCX)

S10 Table Summary of repeat elements, over-represented (based on ‘gene counts’) in the neighborhood of 9 human transcription factor genes. (DOCX)

S11 Table Summary of repeat elements, under-represented (based on ‘gene counts’) in the neighborhood of 9 human transcription factor genes. (DOCX)

S12 Table Summary of repeat elements, over-represented (based on ‘repeat counts’) in the neighborhood of mouse orthologs of 9 human transcription factor genes. (DOCX)

S13 Table Summary of repeat elements, under-represented (based on ‘repeat counts’) in the neighborhood of mouse orthologs of 9 human transcription factor genes. (DOCX)

S14 Table Summary of repeat elements, over-represented (based on ‘gene counts’) in the neighborhood of mouse orthologs of 9 human transcription factor genes. (DOCX)

S15 Table Summary of repeat elements, under-represented (based on ‘gene counts’) in the neighborhood of mouse orthologs of 9 human transcription factor genes. (DOCX)

S16 Table Summary of repeat elements, commonly over-represented (based on ‘repeat counts’) in the neighborhood of 9 human transcription factor genes and their mouse orthologs. (DOCX)

S17 Table Summary of repeat elements, commonly over-represented (based on ‘gene counts’) in the neighborhood of 9 human transcription factor genes and their mouse orthologs. (DOCX)
