# Supplementary material for: GREAM: A Web Server to Short-List Potentially Important Genomic Repeat Elements Based on Over-/Under-Representation in Specific Chromosomal Locations, Such as the Gene Neighborhoods, within or across 17 Mammalian Species
Source: PLoS One. 2015 Jul 24;10(7):e0133647. doi: 10.1371/journal.pone.0133647 (PMC4514817; doi:10.1371/journal.pone.0133647)
Supplement: S1 Table — (DOCX) [file pone.0133647.s001.docx]

**S1 Table.** **Summary of repeat element analysis on human differentially regulated genes of endometrial stromal cells.** Details of the over-represented repeat elements in the neighborhood (200KB either side of the gene) of 1149 genes differentially regulated endometrial stromal cells, with statistical significance of their over-representation is greater than or equal to MER20 (P-value <=0.0234). The analysis was carried out using statistics based on ‘gene counts’.

| **Serial. number** | **Repeat element** | **Repeat class** | **Number genes having MER20 in their neighborhood** | **Observed/Expected ratio** | **P-value** |
| --- | --- | --- | --- | --- | --- |
| 1 | L1MB7 | LINE/L1 | 976 | 1.0804 | <1X 10^-4^ |
| 2 | MIR3 | SINE | 1127 | 1.0219 | <1X 10^-4^ |
| 3 | L1ME4a | LINE/L1 | 1089 | 1.0536 | <1X 10^-4^ |
| 4 | L1MC4 | LINE/L1 | 1023 | 1.0417 | 0.0001 |
| 5 | MLT1D | LTR | 956 | 1.0488 | 0.0001 |
| 6 | GA-rich | Low_complexity | 1086 | 1.0316 | 0.0001 |
| 7 | MLT1A0 | LTR | 942 | 1.0486 | 0.0002 |
| 8 | (A)n | Simple_repeat | 968 | 1.0449 | 0.0002 |
| 9 | L1MC4a | LINE/L1 | 1005 | 1.0397 | 0.0002 |
| 10 | (T)n | Simple_repeat | 964 | 1.0426 | 0.0004 |
| 11 | L1MC5 | LINE/L1 | 978 | 1.0366 | 0.0008 |
| 12 | MER5B | DNA | 1025 | 1.0298 | 0.0012 |
| 13 | MER5A | DNA | 1085 | 1.0215 | 0.0016 |
| 14 | L3 | LINE | 1077 | 1.0206 | 0.0027 |
| 15 | CT-rich | Low_complexity | 1072 | 1.0187 | 0.0048 |
| 16 | L2c | LINE/L2 | 1142 | 1.009 | 0.0052 |
| 17 | L1ME1 | LINE/L1 | 940 | 1.0176 | 0.0146 |
| 18 | FLAM_A | SINE | 1041 | 1.012 | 0.0195 |
| 19 | L2b | LINE/L2 | 1143 | 1.0062 | 0.0207 |
| 20 | FLAM_C | SINE | 1099 | 1.0096 | 0.0214 |
| 21 | MER20 | DNA | 939 | 1.0103 | 0.0234 |
| 22 | MIRc | SINE | 1136 | 1.0069 | 0.0234 |
