# Supplementary material for: GREAM: A Web Server to Short-List Potentially Important Genomic Repeat Elements Based on Over-/Under-Representation in Specific Chromosomal Locations, Such as the Gene Neighborhoods, within or across 17 Mammalian Species
Source: PLoS One. 2015 Jul 24;10(7):e0133647. doi: 10.1371/journal.pone.0133647 (PMC4514817; doi:10.1371/journal.pone.0133647)
Supplement: S2 Table — (DOCX) [file pone.0133647.s002.docx]

**S2 Table. Summary of over-represented repeat elements found within AZFa locus of the human Y chromosome which influence male fertility.**

| **Serial number** | **Repeat element** | **Repeat class** | **Repeat count** | **Observed/Expected ratio** | **P-value** |
| --- | --- | --- | --- | --- | --- |
| 1 | HERV1_LTRc | LTR | 3 | 325.943 | 0 |
| 2 | HERVKC4-int | LTR/ERVK | 3 | 291.6332 | 0 |
| 3 | HERV15-int | LTR/ERV1 | 4 | 152.3308 | 0 |
| 4 | LTR14 | LTR | 3 | 130.3772 | 0 |
| 5 | HERV1_LTRe | LTR | 1 | 119.162 | 0.0083 |
| 6 | LTR10B | LTR | 2 | 92.3505 | 0.0002 |
| 7 | LTR19-int | LTR | 6 | 91.5873 | 0 |
| 8 | LTR21A | LTR | 2 | 79.4413 | 0.0003 |
| 9 | MER61F | LTR | 3 | 74.3763 | 0 |
| 10 | MER51-int | LTR | 10 | 66.2011 | 0 |
| 11 | PABL_A-int | LTR | 10 | 59.6772 | 0 |
| 12 | HERV3-int | LTR/ERV1 | 5 | 59.1991 | 0 |
| 13 | L1M3f | LINE/L1 | 9 | 46.9579 | 0 |
| 14 | LTR25-int | LTR | 7 | 44.4298 | 0 |
| 15 | LTR15 | LTR | 3 | 41.9775 | 0.0001 |
| 16 | Harlequin-int | LTR/ERV1 | 6 | 39.0213 | 0 |
| 17 | MER66-int | LTR | 8 | 37.8389 | 0 |
| 18 | L1MDb | LINE/L1 | 12 | 37.5982 | 0 |
| 19 | LTR19C | LTR | 2 | 35.691 | 0.0015 |
| 20 | LTR2B | LTR | 3 | 34.6314 | 0.0001 |
| 21 | MER88 | LTR | 1 | 33.2795 | 0.0292 |
| 22 | LTR10G | LTR | 1 | 31.5728 | 0.0307 |
| 23 | L1M3c | LINE/L1 | 10 | 31.5728 | 0 |
| 24 | HUERS-P2-int | LTR/ERV1 | 2 | 29.5522 | 0.0021 |
| 25 | tRNA-Cys-TGY | tRNA | 1 | 28.6358 | 0.0337 |
| 26 | MER50-int | LTR | 4 | 28.5253 | 0 |
| 27 | LTR19A | LTR | 3 | 26.1987 | 0.0002 |
| 28 | LTR10F | LTR | 3 | 25.8927 | 0.0002 |
| 29 | L1M4c | LINE/L1 | 42 | 22.9918 | 0 |
| 30 | HERVK9-int | LTR/ERVK | 4 | 22.628 | 0 |
| 31 | MER70B | LTR | 2 | 20.0762 | 0.0045 |
| 32 | L1M3a | LINE/L1 | 3 | 17.5627 | 0.0007 |
| 33 | L1MB4 | LINE/L1 | 41 | 16.4643 | 0 |
| 34 | HUERS-P3b-int | LTR/ERV1 | 2 | 16.3815 | 0.0066 |
| 35 | LTR13 | LTR | 2 | 15.4886 | 0.0073 |
| 36 | MER51B | LTR | 3 | 15.4562 | 0.001 |
| 37 | MER9B | LTR | 5 | 15.2394 | 0 |
| 38 | MER4-int | LTR | 11 | 14.6009 | 0 |
| 39 | MER52-int | LTR | 2 | 14.5148 | 0.0083 |
| 40 | MER21-int | LTR | 4 | 14.5006 | 0.0002 |
| 41 | Tigger4 | DNA | 6 | 13.4328 | 0 |
| 42 | L1P4a | LINE/L1 | 2 | 12.2725 | 0.0113 |
| 43 | L1MCa | LINE/L1 | 25 | 12.253 | 0 |
| 44 | L1PA15-16 | LINE/L1 | 5 | 12.0878 | 0.0001 |
| 45 | MER54A | LTR | 2 | 11.4013 | 0.0129 |
| 46 | L1MA4A | LINE/L1 | 18 | 10.4351 | 0 |
| 47 | MER34A1 | LTR | 2 | 10.0245 | 0.0163 |
| 48 | L1M3e | LINE/L1 | 2 | 9.8771 | 0.0167 |
| 49 | MER4A1 | LTR | 6 | 9.7898 | 0 |
| 50 | LTR9 | LTR | 5 | 9.4187 | 0.0002 |
| 51 | Tigger7 | DNA | 7 | 9.4167 | 0 |
| 52 | L1P2 | LINE/L1 | 4 | 9.0318 | 0.001 |
| 53 | L1M1 | LINE/L1 | 24 | 8.6697 | 0 |
| 54 | LTR2 | LTR | 2 | 8.5018 | 0.0219 |
| 55 | LTR47A | LTR | 2 | 8.3012 | 0.0228 |
| 56 | Tigger2 | DNA | 8 | 8.0436 | 0 |
| 57 | L1PB4 | LINE/L1 | 16 | 7.7301 | 0 |
| 58 | L1M2 | LINE/L1 | 19 | 7.3218 | 0 |
| 59 | Tigger2b_Pri | DNA | 2 | 6.638 | 0.0336 |
| 60 | L1P3 | LINE/L1 | 6 | 6.1126 | 0.0005 |
| 61 | Charlie24 | DNA | 2 | 6.0607 | 0.0391 |
| 62 | MER82 | DNA | 5 | 5.7486 | 0.0017 |
| 63 | ERVL-B4-int | LTR/ERVL | 5 | 5.5734 | 0.002 |
| 64 | L1MD1 | LINE/L1 | 11 | 5.5595 | 0 |
| 65 | MER52A | LTR | 3 | 5.3024 | 0.0171 |
| 66 | L1MB5 | LINE/L1 | 13 | 4.9138 | 0 |
| 67 | L1MCb | LINE/L1 | 3 | 4.527 | 0.025 |
| 68 | MSTB1 | LTR | 6 | 4.4668 | 0.0021 |
| 69 | AluJ/FLAM | SINE/Alu | 4 | 4.3319 | 0.012 |
| 70 | L1M3 | LINE/L1 | 8 | 4.2552 | 0.0006 |
| 71 | L1MDa | LINE/L1 | 8 | 4.1693 | 0.0007 |
| 72 | L1MB3 | LINE/L1 | 19 | 3.996 | 0 |
| 73 | AluYa5 | SINE | 4 | 3.8053 | 0.0178 |
| 74 | L1P4 | LINE/L1 | 4 | 3.6394 | 0.0202 |
| 75 | L1PA16 | LINE/L1 | 14 | 3.5987 | 0 |
| 76 | L1PA11 | LINE/L1 | 4 | 3.5665 | 0.0214 |
| 77 | L1M4 | LINE/L1 | 17 | 3.4832 | 0 |
| 78 | L1PA15 | LINE/L1 | 8 | 3.4411 | 0.0021 |
| 79 | L1PA6 | LINE/L1 | 5 | 3.1126 | 0.0178 |
| 80 | L1MB2 | LINE/L1 | 7 | 2.8183 | 0.0096 |
| 81 | L1PA10 | LINE/L1 | 5 | 2.5288 | 0.0348 |
| 82 | L1MA9 | LINE/L1 | 10 | 2.1841 | 0.0114 |
| 83 | Tigger1 | DNA | 7 | 2.0233 | 0.0369 |
| 84 | L1PA7 | LINE/L1 | 7 | 1.9538 | 0.0417 |
| 85 | AluSc | SINE | 24 | 1.7831 | 0.0028 |
| 86 | L1MC4a | LINE/L1 | 12 | 1.718 | 0.0259 |
| 87 | AluSq | SINE | 41 | 1.6081 | 0.0011 |
| 88 | AluSg | SINE | 35 | 1.5744 | 0.0028 |
| 89 | AluY | SINE | 47 | 1.262 | 0.0176 |
| 90 | AluSx | SINE | 114 | 1.2416 | 0.0027 |
| 91 | AluJb | SINE | 42 | 1.1943 | 0.0328 |
