# Supplementary material for: GREAM: A Web Server to Short-List Potentially Important Genomic Repeat Elements Based on Over-/Under-Representation in Specific Chromosomal Locations, Such as the Gene Neighborhoods, within or across 17 Mammalian Species
Source: PLoS One. 2015 Jul 24;10(7):e0133647. doi: 10.1371/journal.pone.0133647 (PMC4514817; doi:10.1371/journal.pone.0133647)
Supplement: S3 Table — (DOCX) [file pone.0133647.s003.docx]

**S3 Table. Summary of under-represented repeat elements found within AZFa locus of the human Y chromosome which influence male fertility.**

| **Serial number** | **Repeat element** | **Repeat class** | **Repeat count** | **Observed/Expected ratio** | **P-value** |
| --- | --- | --- | --- | --- | --- |
| 1 | AT_rich | Low_complexity | 73 | 0.9292 | 0.0387 |
| 2 | AluJo | SINE | 28 | 0.7346 | 0.0167 |
| 3 | MER5A | DNA | 4 | 0.4276 | 0.0274 |
| 4 | HAL1 | LINE | 3 | 0.405 | 0.0409 |
| 5 | L1M5 | LINE/L1 | 6 | 0.3805 | 0.0029 |
| 6 | (TG)n | Simple_repeat | 5 | 0.3494 | 0.003 |
| 7 | GA-rich | Low_complexity | 2 | 0.3185 | 0.0368 |
| 8 | MER5B | DNA | 2 | 0.2965 | 0.0266 |
| 9 | MIR | SINE | 15 | 0.2844 | 0 |
| 10 | L1ME3C | LINE/L1 | 1 | 0.1944 | 0.0298 |
| 11 | MLT1C | LTR | 1 | 0.1855 | 0.0244 |
| 12 | MLT1A0 | LTR | 1 | 0.1787 | 0.0206 |
| 13 | L2a | LINE/L2 | 8 | 0.1751 | 0 |
| 14 | MIRb | SINE | 11 | 0.1746 | 0 |
| 15 | THE1B | LTR | 1 | 0.1653 | 0.0142 |
| 16 | L1MB7 | LINE/L1 | 1 | 0.1591 | 0.0116 |
| 17 | MIR3 | SINE | 2 | 0.1097 | 0 |
| 18 | L2c | LINE/L2 | 4 | 0.1071 | 0 |
| 19 | L3 | LINE | 1 | 0.0865 | 0.0001 |
| 20 | MIRc | SINE | 2 | 0.0822 | 0 |
