# Supplementary material for: GREAM: A Web Server to Short-List Potentially Important Genomic Repeat Elements Based on Over-/Under-Representation in Specific Chromosomal Locations, Such as the Gene Neighborhoods, within or across 17 Mammalian Species
Source: PLoS One. 2015 Jul 24;10(7):e0133647. doi: 10.1371/journal.pone.0133647 (PMC4514817; doi:10.1371/journal.pone.0133647)
Supplement: S4 Table — (DOCX) [file pone.0133647.s004.docx]

**S4 Table. Summary of repeat elements, over-represented (based on ‘repeat counts’) in the neighborhood of 64 rat genes associated with general rat injury.**

| **Serial number** | **Repeat element** | **Repeat class** | **Repeat count** | **Observed/Expected ratio** | **P-value** |
| --- | --- | --- | --- | --- | --- |
| 1 | ERVB1_2-LTR_RN | LTR | 6 | 254.3569 | 0 |
| 2 | MER110-int | LTR | 1 | 127.1784 | 0.0078 |
| 3 | Zaphod2 | DNA | 5 | 68.7451 | 0 |
| 4 | MER34-int | LTR | 3 | 52.6256 | 0 |
| 5 | Ricksha_c | DNA | 4 | 52.1758 | 0 |
| 6 | Charlie17a | DNA | 1 | 31.7946 | 0.0305 |
| 7 | UCON29 | DNA | 1 | 31.7946 | 0.0305 |
| 8 | AmnSINE2 | SINE | 1 | 28.2619 | 0.0342 |
| 9 | X8_LINE | LINE | 1 | 25.4357 | 0.0378 |
| 10 | tRNA-Met_ | tRNA | 1 | 24.2245 | 0.0396 |
| 11 | MER57E1 | LTR | 2 | 22.118 | 0.0037 |
| 12 | (CAGCT)n | Simple_repeat | 1 | 19.5659 | 0.0486 |
| 13 | MER67B | LTR | 3 | 18.6115 | 0.0006 |
| 14 | MER57-int | LTR | 2 | 18.4987 | 0.0052 |
| 15 | RLTR18-int | LTR | 5 | 16.0985 | 0 |
| 16 | (CAGC)n | Simple_repeat | 2 | 14.33 | 0.0085 |
| 17 | MER57C2 | LTR | 2 | 11.059 | 0.0136 |
| 18 | RNLTR21 | LTR | 5 | 10.5106 | 0.0001 |
| 19 | RLTR31_Mur | LTR | 4 | 10.0735 | 0.0007 |
| 20 | RatERVL-int | LTR/ERVL | 7 | 9.7562 | 0 |
| 21 | RLTR30 | LTR | 3 | 9.5984 | 0.0037 |
| 22 | MER90a | LTR | 3 | 9.5384 | 0.0038 |
| 23 | RNLTR10A | LTR | 4 | 9.3342 | 0.0009 |
| 24 | RNLTR20 | LTR | 2 | 8.8472 | 0.0204 |
| 25 | RNLTR21-int | LTR | 3 | 8.4786 | 0.0052 |
| 26 | ORR1E-int | LTR/ERVL-MaLR | 5 | 8.0493 | 0.0004 |
| 27 | (GGGGA)n | Simple_repeat | 2 | 7.2673 | 0.0288 |
| 28 | MER115 | DNA | 2 | 6.7829 | 0.0324 |
| 29 | MER117 | DNA | 2 | 6.564 | 0.0342 |
| 30 | ORR1B1-int | LTR | 8 | 6.2804 | 0 |
| 31 | L1ME3D | LINE/L1 | 2 | 6.1662 | 0.038 |
| 32 | MER68 | LTR | 2 | 5.8139 | 0.0419 |
| 33 | MT2_Rat2 | LTR | 4 | 5.7159 | 0.005 |
| 34 | Tigger5 | DNA | 2 | 5.6211 | 0.0443 |
| 35 | Charlie1b | DNA | 3 | 5.3927 | 0.0164 |
| 36 | RLTR20C | LTR | 4 | 5.0871 | 0.0072 |
| 37 | L1M4b | LINE/L1 | 3 | 4.0589 | 0.0321 |
| 38 | RMER6C | LTR | 5 | 3.9681 | 0.0075 |
| 39 | RLTR11A2 | LTR | 6 | 3.7729 | 0.0046 |
| 40 | MERVL_2A-int | LTR/ERVL | 5 | 3.1914 | 0.0164 |
| 41 | RMER3-int | LTR | 4 | 3.0146 | 0.0342 |
| 42 | RMER19C | LTR | 7 | 2.9725 | 0.0075 |
| 43 | (CAGAGA)n | Simple_repeat | 8 | 2.846 | 0.0058 |
| 44 | ORR1B1 | LTR | 17 | 2.4773 | 0.0005 |
| 45 | L2c | LINE/L2 | 13 | 2.4216 | 0.0023 |
| 46 | (CCA)n | Simple_repeat | 6 | 2.4091 | 0.0274 |
| 47 | L1MC4 | LINE/L1 | 6 | 2.379 | 0.0287 |
| 48 | MER20 | DNA | 7 | 2.2989 | 0.0229 |
| 49 | MTEa | LTR | 16 | 2.1769 | 0.0022 |
| 50 | Lx10 | LINE/L1 | 7 | 2.1595 | 0.0291 |
| 51 | ORR1B2 | LTR | 7 | 2.0752 | 0.0338 |
| 52 | L2a | LINE/L2 | 20 | 1.8123 | 0.0047 |
| 53 | (T)n | Simple_repeat | 20 | 1.7887 | 0.0053 |
| 54 | MIRc | SINE | 14 | 1.7477 | 0.017 |
| 55 | CT-rich | Low_complexity | 29 | 1.7051 | 0.0022 |
| 56 | (GA)n | Simple_repeat | 39 | 1.6357 | 0.0011 |
| 57 | ID4_ | SINE | 27 | 1.6197 | 0.0051 |
| 58 | (A)n | Simple_repeat | 15 | 1.4147 | 0.0457 |
| 59 | ID4 | SINE | 23 | 1.3924 | 0.0266 |
| 60 | MIR | SINE | 29 | 1.3199 | 0.0265 |
| 61 | ID_Rn2 | SINE | 67 | 1.2475 | 0.0104 |
| 62 | MIRb | SINE | 26 | 1.2231 | 0.0476 |
| 63 | PB1D10 | SINE | 66 | 1.2117 | 0.0154 |
| 64 | B4A | SINE | 62 | 1.1716 | 0.0241 |
| 65 | (TG)n | Simple_repeat | 73 | 1.0037 | 0.0473 |
