# Supplementary material for: GREAM: A Web Server to Short-List Potentially Important Genomic Repeat Elements Based on Over-/Under-Representation in Specific Chromosomal Locations, Such as the Gene Neighborhoods, within or across 17 Mammalian Species
Source: PLoS One. 2015 Jul 24;10(7):e0133647. doi: 10.1371/journal.pone.0133647 (PMC4514817; doi:10.1371/journal.pone.0133647)
Supplement: S5 Table — (DOCX) [file pone.0133647.s005.docx]

**S5 Table. Summary of repeat elements, under-represented (based on ‘repeat counts’) in the neighborhood of 64 rat genes associated with general rat injury.**

| **Serial number** | **Repeat element** | **Repeat class** | **Repeat count** | **Observed/Expected ratio** | **P-value** |
| --- | --- | --- | --- | --- | --- |
| 1 | B3 | SINE | 97 | 0.9669 | 0.039 |
| 2 | RSINE1 | SINE | 73 | 0.9515 | 0.0431 |
| 3 | (CA)n | Simple_repeat | 69 | 0.9501 | 0.0443 |
| 4 | ID_B1 | SINE | 86 | 0.9481 | 0.0385 |
| 5 | AT_rich | Low_complexity | 82 | 0.8809 | 0.0219 |
| 6 | B1_Rn | SINE | 43 | 0.8715 | 0.0397 |
| 7 | B1_Mur3 | SINE | 17 | 0.6972 | 0.0273 |
| 8 | PB1D7 | SINE | 14 | 0.6828 | 0.033 |
| 9 | MTC | LTR | 7 | 0.5507 | 0.0319 |
| 10 | L1_Rat4 | LINE/L1 | 7 | 0.5462 | 0.0305 |
| 11 | L1_Rn | LINE/L1 | 6 | 0.4751 | 0.0183 |
| 12 | L1_Rn2 | LINE/L1 | 5 | 0.4473 | 0.0202 |
| 13 | L1M2 | LINE/L1 | 3 | 0.4127 | 0.0445 |
| 14 | Lx5 | LINE/L1 | 3 | 0.2781 | 0.0043 |
| 15 | Lx | LINE/L1 | 2 | 0.1666 | 0.0004 |
| 16 | Lx8b | LINE/L1 | 1 | 0.1382 | 0.0052 |
