# Supplementary material for: GREAM: A Web Server to Short-List Potentially Important Genomic Repeat Elements Based on Over-/Under-Representation in Specific Chromosomal Locations, Such as the Gene Neighborhoods, within or across 17 Mammalian Species
Source: PLoS One. 2015 Jul 24;10(7):e0133647. doi: 10.1371/journal.pone.0133647 (PMC4514817; doi:10.1371/journal.pone.0133647)
Supplement: S6 Table — (DOCX) [file pone.0133647.s006.docx]

**S6 Table. Summary of repeat elements, over-represented (based on ‘gene counts’) in the neighborhood of 64 rat genes associated with general rat injury.**

| **Serial number** | **Repeat element** | **Repeat class** | **Gene count** | **Observed/Expected ratio** | **P-value** |
| --- | --- | --- | --- | --- | --- |
| 1 | MER110-int | LTR | 1 | 133.8178 | 0.0074 |
| 2 | ERVB1_2-LTR_RN | LTR | 1 | 76.4673 | 0.0129 |
| 3 | MER34-int | LTR | 1 | 38.2337 | 0.0255 |
| 4 | UCON29 | DNA | 1 | 38.2337 | 0.0255 |
| 5 | (CAGCT)n | Simple_repeat | 1 | 33.4544 | 0.029 |
| 6 | Charlie17a | DNA | 1 | 33.4544 | 0.029 |
| 7 | tRNA-Met_ | tRNA | 1 | 33.4544 | 0.029 |
| 8 | AmnSINE2 | SINE | 1 | 29.7373 | 0.0325 |
| 9 | X8_LINE | LINE | 1 | 26.7636 | 0.036 |
| 10 | L1M3de | LINE/L1 | 1 | 24.3305 | 0.0395 |
| 11 | Zaphod2 | DNA | 1 | 21.4108 | 0.0446 |
| 12 | MER67B | LTR | 2 | 18.7814 | 0.005 |
| 13 | (CAGC)n | Simple_repeat | 2 | 15.0781 | 0.0076 |
| 14 | RNLTR21-int | LTR | 2 | 11.3887 | 0.0128 |
| 15 | RNLTR20 | LTR | 2 | 10.7054 | 0.0143 |
| 16 | RLTR31_Mur | LTR | 3 | 9.2288 | 0.004 |
| 17 | MER90a | LTR | 2 | 8.5643 | 0.0214 |
| 18 | RLTR30 | LTR | 2 | 8.2349 | 0.0229 |
| 19 | RNLTR21 | LTR | 3 | 7.9104 | 0.006 |
| 20 | (GGGGA)n | Simple_repeat | 2 | 7.6467 | 0.0261 |
| 21 | L1ME3D | LINE/L1 | 2 | 7.4343 | 0.0274 |
| 22 | MTEa-int | LTR/ERVL-MaLR | 2 | 7.3831 | 0.0277 |
| 23 | MER117 | DNA | 2 | 7.2826 | 0.0284 |
| 24 | RLTR17 | LTR | 2 | 5.5469 | 0.0451 |
| 25 | ORR1B1-int | LTR | 4 | 4.9793 | 0.0074 |
| 26 | MERVL_2A-int | LTR/ERVL | 3 | 4.4359 | 0.0257 |
| 27 | RLTR11A2 | LTR | 4 | 3.2343 | 0.0274 |
| 28 | (CAGAGA)n | Simple_repeat | 7 | 2.869 | 0.0078 |
| 29 | (CCA)n | Simple_repeat | 6 | 2.7639 | 0.0153 |
| 30 | L2c | LINE/L2 | 11 | 2.5434 | 0.0024 |
| 31 | ORR1B2 | LTR | 6 | 2.4039 | 0.0261 |
| 32 | (TTTTTG)n | Simple_repeat | 5 | 2.2777 | 0.0461 |
| 33 | MTEa | LTR | 12 | 2.218 | 0.0043 |
| 34 | (T)n | Simple_repeat | 16 | 1.7921 | 0.0072 |
| 35 | ID4_ | SINE | 22 | 1.7469 | 0.0022 |
| 36 | MIRc | SINE | 11 | 1.7353 | 0.0263 |
| 37 | L2a | LINE/L2 | 13 | 1.7089 | 0.0192 |
| 38 | (A)n | Simple_repeat | 13 | 1.5247 | 0.037 |
| 39 | MIR | SINE | 23 | 1.5227 | 0.0084 |
| 40 | PB1 | SINE | 12 | 1.4831 | 0.0483 |
| 41 | CT-rich | Low_complexity | 19 | 1.4665 | 0.0213 |
| 42 | (GA)n | Simple_repeat | 25 | 1.3941 | 0.0159 |
| 43 | ID4 | SINE | 17 | 1.3563 | 0.0447 |
| 44 | PB1D10 | SINE | 36 | 1.2867 | 0.0118 |
| 45 | ID_Rn2 | SINE | 33 | 1.1861 | 0.0419 |
