# Supplementary material for: GREAM: A Web Server to Short-List Potentially Important Genomic Repeat Elements Based on Over-/Under-Representation in Specific Chromosomal Locations, Such as the Gene Neighborhoods, within or across 17 Mammalian Species
Source: PLoS One. 2015 Jul 24;10(7):e0133647. doi: 10.1371/journal.pone.0133647 (PMC4514817; doi:10.1371/journal.pone.0133647)
Supplement: S7 Table — (DOCX) [file pone.0133647.s007.docx]

**S7 Table. Summary of repeat elements, under-represented (based on ‘gene counts’) in the neighborhood of 64 rat genes associated with general rat injury.**

| **Serial number** | **Repeat element** | **Repeat class** | **Gene count** | **Observed/Expected ratio** | **P-value** |
| --- | --- | --- | --- | --- | --- |
| 1 | Lx | LINE/L1 | 2 | 0.2647 | 0.0114 |
| 2 | Lx8b | LINE/L1 | 1 | 0.2147 | 0.0395 |
| 3 | Lx5 | LINE/L1 | 1 | 0.1588 | 0.009 |
