# Supplementary material for: GREAM: A Web Server to Short-List Potentially Important Genomic Repeat Elements Based on Over-/Under-Representation in Specific Chromosomal Locations, Such as the Gene Neighborhoods, within or across 17 Mammalian Species
Source: PLoS One. 2015 Jul 24;10(7):e0133647. doi: 10.1371/journal.pone.0133647 (PMC4514817; doi:10.1371/journal.pone.0133647)
Supplement: S8 Table — (DOCX) [file pone.0133647.s008.docx]

**S8 Table. Summary of repeat elements, over-represented (based on ‘repeat counts’) in the neighborhood of 9 human transcription factor genes.**

| **Serial number** | **Repeat element** | **Repeat class** | **Repeat count** | **Observed/Expected ratio** | **P-value** |
| --- | --- | --- | --- | --- | --- |
| 1 | Charlie25 | DNA | 4 | 156.607 | 0 |
| 2 | (CCGGG)n | Simple_repeat | 1 | 64.249 | 0.0153 |
| 3 | L1P2 | LINE/L1 | 2 | 59.19 | 0.0005 |
| 4 | MER39B | LTR | 2 | 43.9599 | 0.001 |
| 5 | (CAGA)n | Simple_repeat | 1 | 41.9952 | 0.0233 |
| 6 | Tigger8 | DNA | 1 | 35.2917 | 0.0275 |
| 7 | Charlie16a | DNA | 2 | 27.485 | 0.0025 |
| 8 | L1MEe | LINE/L1 | 8 | 24.809 | 0 |
| 9 | (GGGGA)n | Simple_repeat | 1 | 24.2488 | 0.0396 |
| 10 | MER9B | LTR | 1 | 23.2729 | 0.0412 |
| 11 | MLT1G1 | LTR | 2 | 22.3724 | 0.0036 |
| 12 | (CTGGGG)n | Simple_repeat | 1 | 21.175 | 0.0451 |
| 13 | L1PA8A | LINE/L1 | 1 | 19.3243 | 0.0492 |
| 14 | (TTCC)n | Simple_repeat | 2 | 18.6761 | 0.0051 |
| 15 | C-rich | Low_complexity | 5 | 13.9 | 0 |
| 16 | L1PA6 | LINE/L1 | 2 | 13.4595 | 0.0095 |
| 17 | L1PA5 | LINE/L1 | 3 | 12.6409 | 0.0017 |
| 18 | L1MD | LINE/L1 | 3 | 10.4988 | 0.0029 |
| 19 | G-rich | Low_complexity | 3 | 7.8879 | 0.0062 |
| 20 | CT-rich | Low_complexity | 5 | 6.3254 | 0.0011 |
| 21 | L2b | LINE/L2 | 11 | 3.3037 | 0.0004 |
| 22 | L2 | LINE | 5 | 2.6061 | 0.0314 |
| 23 | GC_rich | Low_complexity | 5 | 2.2387 | 0.0493 |
| 24 | MIRb | SINE | 13 | 1.7044 | 0.0221 |
