# Supplementary material for: GREAM: A Web Server to Short-List Potentially Important Genomic Repeat Elements Based on Over-/Under-Representation in Specific Chromosomal Locations, Such as the Gene Neighborhoods, within or across 17 Mammalian Species
Source: PLoS One. 2015 Jul 24;10(7):e0133647. doi: 10.1371/journal.pone.0133647 (PMC4514817; doi:10.1371/journal.pone.0133647)
Supplement: S10 Table — (DOCX) [file pone.0133647.s010.docx]

**S10 Table. Summary of repeat elements, over-represented (based on ‘gene counts’) in the neighborhood of 9 human transcription factor genes.**

| **Serial number** | **Repeat element** | **Repeat class** | **Gene count** | **Observed/Expected ratio** | **P-value** |
| --- | --- | --- | --- | --- | --- |
| 1 | Charlie25 | DNA | 1 | 47.8301 | 0.0205 |
| 2 | (CCGGG)n | Simple_repeat | 1 | 33.5996 | 0.029 |
| 3 | Tigger8 | DNA | 1 | 25.7314 | 0.0375 |
| 4 | (CAGA)n | Simple_repeat | 1 | 24.3446 | 0.0396 |
| 5 | L1P2 | LINE/L1 | 1 | 19.7357 | 0.0484 |
| 6 | Charlie16a | DNA | 2 | 16.0061 | 0.0063 |
| 7 | (TTCC)n | Simple_repeat | 2 | 10.5189 | 0.0138 |
| 8 | L1PA6 | LINE/L1 | 2 | 9.0446 | 0.0183 |
| 9 | C-rich | Low_complexity | 4 | 7.0705 | 0.0014 |
| 10 | L1MD | LINE/L1 | 2 | 6.0053 | 0.0379 |
| 11 | L1PA5 | LINE/L1 | 2 | 5.5164 | 0.0438 |
| 12 | G-rich | Low_complexity | 3 | 4.8554 | 0.0177 |
| 13 | CT-rich | Low_complexity | 5 | 4.0566 | 0.0034 |
