# Supplementary material for: GREAM: A Web Server to Short-List Potentially Important Genomic Repeat Elements Based on Over-/Under-Representation in Specific Chromosomal Locations, Such as the Gene Neighborhoods, within or across 17 Mammalian Species
Source: PLoS One. 2015 Jul 24;10(7):e0133647. doi: 10.1371/journal.pone.0133647 (PMC4514817; doi:10.1371/journal.pone.0133647)
Supplement: S11 Table — (DOCX) [file pone.0133647.s011.docx]

**S11 Table. Summary of repeat elements, under-represented (based on ‘gene counts’) in the neighborhood of 9 human transcription factor genes.**

| **Serial number** | **Repeat element** | **Repeat class** | **Gene count** | **Observed/Expected ratio** | **P-value** |
| --- | --- | --- | --- | --- | --- |
| 1 | AluSx | SINE | 4 | 0.5394 | 0.0098 |
| 2 | AT_rich | Low_complexity | 3 | 0.4781 | 0.0219 |
| 3 | AluJo | SINE | 2 | 0.3772 | 0.0247 |
| 4 | AluJb | SINE | 2 | 0.3726 | 0.0223 |
| 5 | AluSq | SINE | 1 | 0.2144 | 0.0135 |
