# Supplementary material for: GREAM: A Web Server to Short-List Potentially Important Genomic Repeat Elements Based on Over-/Under-Representation in Specific Chromosomal Locations, Such as the Gene Neighborhoods, within or across 17 Mammalian Species
Source: PLoS One. 2015 Jul 24;10(7):e0133647. doi: 10.1371/journal.pone.0133647 (PMC4514817; doi:10.1371/journal.pone.0133647)
Supplement: S12 Table — (DOCX) [file pone.0133647.s012.docx]

**S12 Table. Summary of repeat elements, over-represented (based on repeat counts) in the neighborhood of mouse orthologs of 9 human transcription factor genes.**

| **Serial number** | **Repeat element** | **Repeat class** | **Repeat count** | **Observed/Expected ratio** | **P-value** |
| --- | --- | --- | --- | --- | --- |
| 1 | L1M2b | LINE/L1 | 1 | 1237.24 | 0.0008 |
| 2 | (TTAGGC)n | Simple_repeat | 1 | 1031.034 | 0.001 |
| 3 | (TTCGGG)n | Simple_repeat | 1 | 475.8617 | 0.0021 |
| 4 | MLT1G | LTR | 3 | 93.7303 | 0 |
| 5 | (CACAC)n | Simple_repeat | 1 | 65.1179 | 0.0151 |
| 6 | MamRep605 | Unknown | 1 | 39.4026 | 0.0247 |
| 7 | ORR1D2-int | LTR/ERVL-MaLR | 2 | 35.451 | 0.0015 |
| 8 | L1MD3 | LINE/L1 | 2 | 27.5555 | 0.0024 |
| 9 | (CACCAT)n | Simple_repeat | 1 | 25.1472 | 0.0382 |
| 10 | (GGGAGA)n | Simple_repeat | 2 | 23.7474 | 0.0032 |
| 11 | (CCCCCT)n | Simple_repeat | 1 | 20.2163 | 0.0471 |
| 12 | Charlie1b | DNA | 1 | 19.0345 | 0.0499 |
| 13 | L1MD | LINE/L1 | 3 | 14.2868 | 0.0012 |
| 14 | L3 | LINE | 4 | 13.641 | 0.0002 |
| 15 | L1MC1 | LINE/L1 | 2 | 10.01 | 0.0163 |
| 16 | L1M3 | LINE/L1 | 2 | 7.4758 | 0.0273 |
| 17 | C-rich | Low_complexity | 3 | 7.2297 | 0.0078 |
| 18 | L1M5 | LINE/L1 | 4 | 7.21 | 0.0022 |
| 19 | G-rich | Low_complexity | 3 | 6.9017 | 0.0088 |
| 20 | GC_rich | Low_complexity | 10 | 5.8698 | 0 |
| 21 | L2 | LINE | 4 | 5.6392 | 0.0051 |
| 22 | (A)n | Simple_repeat | 7 | 4.0756 | 0.0015 |
| 23 | PB1D7 | SINE | 5 | 2.6347 | 0.0305 |
| 24 | AT_rich | Low_complexity | 22 | 2.2929 | 0.0002 |
| 25 | (CA)n | Simple_repeat | 12 | 1.8406 | 0.0175 |
