# Supplementary material for: GREAM: A Web Server to Short-List Potentially Important Genomic Repeat Elements Based on Over-/Under-Representation in Specific Chromosomal Locations, Such as the Gene Neighborhoods, within or across 17 Mammalian Species
Source: PLoS One. 2015 Jul 24;10(7):e0133647. doi: 10.1371/journal.pone.0133647 (PMC4514817; doi:10.1371/journal.pone.0133647)
Supplement: S14 Table — (DOCX) [file pone.0133647.s014.docx]

**S14 Table. Summary of repeat elements, over-represented (based on ‘gene counts’) in the neighborhood of mouse orthologs of 9 human transcription factor genes.**

| **Serial number** | **Repeat element** | **Repeat class** | **Gene count** | **Observed/Expected ratio** | **P-value** |
| --- | --- | --- | --- | --- | --- |
| 1 | L1M2b | LINE/L1 | 1 | 1000.056 | 0.001 |
| 2 | (TTAGGC)n | Simple_repeat | 1 | 666.7037 | 0.0015 |
| 3 | (TTCGGG)n | Simple_repeat | 1 | 400.0222 | 0.0025 |
| 4 | (CACAC)n | Simple_repeat | 1 | 49.3855 | 0.0199 |
| 5 | ORR1D2-int | LTR/ERVL-MaLR | 2 | 41.0279 | 0.001 |
| 6 | MamRep605 | Unknown | 1 | 27.7793 | 0.0349 |
| 7 | MLT1G | LTR | 1 | 23.8108 | 0.0405 |
| 8 | (GGGAGA)n | Simple_repeat | 2 | 15.6871 | 0.0065 |
| 9 | L3 | LINE | 3 | 7.238 | 0.0062 |
| 10 | C-rich | Low_complexity | 3 | 5.1351 | 0.0154 |
| 11 | G-rich | Low_complexity | 3 | 4.8763 | 0.0175 |
| 12 | L2 | LINE | 4 | 4.6487 | 0.0064 |
| 13 | GC_rich | Low_complexity | 6 | 3.4719 | 0.0022 |
