# Supplementary material for: GREAM: A Web Server to Short-List Potentially Important Genomic Repeat Elements Based on Over-/Under-Representation in Specific Chromosomal Locations, Such as the Gene Neighborhoods, within or across 17 Mammalian Species
Source: PLoS One. 2015 Jul 24;10(7):e0133647. doi: 10.1371/journal.pone.0133647 (PMC4514817; doi:10.1371/journal.pone.0133647)
Supplement: S15 Table — (DOCX) [file pone.0133647.s015.docx]

**S15 Table. Summary of repeat elements, under-represented (based on ‘gene counts’) in the neighborhood of mouse orthologs of 9 human transcription factor genes.**

| **Serial number** | **Repeat element** | **Repeat class** | **Gene count** | **Observed/Expected ratio** | **P-value** |
| --- | --- | --- | --- | --- | --- |
| 1 | B3 | SINE | 3 | 0.5096 | 0.0403 |
| 2 | B1_Mus1 | SINE | 2 | 0.3948 | 0.0348 |
| 3 | B1_Mus2 | SINE | 1 | 0.2376 | 0.0271 |
| 4 | B2_Mm2 | SINE | 1 | 0.218 | 0.0153 |
