# Supplementary material for: GREAM: A Web Server to Short-List Potentially Important Genomic Repeat Elements Based on Over-/Under-Representation in Specific Chromosomal Locations, Such as the Gene Neighborhoods, within or across 17 Mammalian Species
Source: PLoS One. 2015 Jul 24;10(7):e0133647. doi: 10.1371/journal.pone.0133647 (PMC4514817; doi:10.1371/journal.pone.0133647)
Supplement: S16 Table — (DOCX) [file pone.0133647.s016.docx]

**S16 Table. Summary of repeat elements, commonly over-represented (based on ‘repeat counts’) in the neighborhood of 9 human transcription factor genes and their mouse orthologs.**

| **Serial number** | **Repeat element** | **Repeat class** | **Repeat count (human gene-set)** | **Observed/Expected ratio (human gene-set)** | **P-value (human gene-set)** | **Repeat count (mouse gene-set)** | **Observed/Expected ratio (mouse gene-set)** | **P-value (mouse gene-set)** |
| --- | --- | --- | --- | --- | --- | --- | --- | --- |
| 1 | L1MD | LINE/L1 | 3 | 10.4988 | 0.0029 | 3 | 14.2868 | 0.0012 |
| 2 | G-rich | Low_complexity | 3 | 7.8879 | 0.0062 | 3 | 6.9017 | 0.0088 |
| 3 | C-rich | Low_complexity | 5 | 13.9 | 0 | 3 | 7.2297 | 0.0078 |
| 4 | GC_rich | Low_complexity | 5 | 2.2387 | 0.0493 | 10 | 5.8698 | 0 |
| 5 | L2 | LINE | 5 | 2.6061 | 0.0314 | 4 | 5.6392 | 0.0051 |
