# Supplementary material for: GREAM: A Web Server to Short-List Potentially Important Genomic Repeat Elements Based on Over-/Under-Representation in Specific Chromosomal Locations, Such as the Gene Neighborhoods, within or across 17 Mammalian Species
Source: PLoS One. 2015 Jul 24;10(7):e0133647. doi: 10.1371/journal.pone.0133647 (PMC4514817; doi:10.1371/journal.pone.0133647)
Supplement: S17 Table — (DOCX) [file pone.0133647.s017.docx]

**S17 Table. Summary of repeat elements, commonly over-represented (based on ‘gene counts’) in the neighborhood of 9 human transcription factor genes and their mouse orthologs.**

| **Serial number** | **Repeat element** | **Repeat class** | **Repeat count (human gene-set)** | **Observed/Expected ratio (human gene-set)** | **P-value (human gene-set)** | **Repeat count (mouse gene-set)** | **Observed/Expected ratio (mouse gene-set)** | **P-value (mouse gene-set)** |
| --- | --- | --- | --- | --- | --- | --- | --- | --- |
| 1 | G-rich | Low_complexity | 3 | 4.8554 | 0.0177 | 3 | 4.8763 | 0.0175 |
| 2 | C-rich | Low_complexity | 4 | 7.0705 | 0.0014 | 3 | 5.1351 | 0.0154 |
